# A Hydrocyanic Acid Case

**Published:** 1897-11

**Authors:** A. S. Ironside

**Affiliations:** 509 Broadway, Camden, N. J.


### A HYDROCYANIC ACID CASE.

509 Broadway, Camden, N. J., September 20th, 1897.

Editor of The Homœopathic Physician:

In the August number of The Homœopathic Physician Dr. Gilbert desires contributions of clinical experience in typhoid troubles, so I forward you an account of a case that was extremely interesting to me. It is a verification of the symptom upon swallowing liquids, "they gurgle and roll audibly from œsophagus into the bowels." It is a positive indication for Hydro-acid, and I have verified once since that case the symptoms of the pupils mentioned in this case, in a case of whooping cough.

If this case is worth reporting in your journal, please do so, for should another physician receive as much satisfaction from the reading of this case as I had in giving it, he will, at least for a time, be a happy man.

The following is the statement of the case:

A boy four years of age commenced crying out with sharp pains in the region of the stomach about February 10th last, accompanied by high fever, red face, and some thirst. He was given Bell., Bry., Sulph., and Lyc. during the next ten days without any perceptible effect, except that the pains gradually disappeared under the two first remedies. He continued quite sick, and began to develop some brain symptoms. I was also told of an injury of the head that happened by falling some months previous. On February 20th he was in the following condition:

Pulse, 132.

Head hot; body cool; limbs, hands, and feet cold.

Face pale; dark circles under the eyes.

Pupils much contracted when eyes were open, or upon lifting one of the eyelids, except when you would arouse him. When upon looking at you the pupils would promptly dilate.

When aroused he would answer "yes" or "no" to a question, then relapse into a state of stupor.

Has spells of crying and raising body upon head and heels.

No thirst.

Urinates once in twenty-four hours. Urine thick reddish brown when passed.

When swallowing a mouthful of water or spoonful of any liquid, it sounds like water rolling into an empty barrel (the mother of the patient remarked this as strange). Sound commences just as soon as water enters œsophagus, and rolls on down into stomach and bowels.

Tongue, heavy coat, brownish white color; some papillæ to be seen through it. No sweat. Asks for nothing. Motion of alæ nasi when breathing.

The peculiar sound upon swallowing was the last symptom observed, and I returned to the office to look it up. Found it under Hydrocyanic-acid, and upon going over this remedy I found it covered his condition in a surprising manner.

He was given the two-hundredth potency in water. Fifteen minutes after administration the body of the child commenced to grow warm, and in two hours he was in a hot fever. This was from 10 to 12 P. M. In the morning his body was warm, pulse 120, and the child was sufficiently intelligent to recognize those in the room, and to follow them with his eyes. Pupils normal size. He had been in the stupor mentioned between three and four days.

In the course of thirty-six or forty-eight hours the crying with pains in the stomach returned for about twelve hours, when spells of crying grew farther apart, and after two days more had a small, hard, light-colored stool. Urinated easily, urine being clear and high in color. He recovered completely.

Fraternally,

A. S. IRONSIDE.

